# Adult presentation of SLC29A3-related disorder spectrum with marked hypogammaglobulinemia due to a recurrent homozygous pathogenic variant (G437R): a case report

**DOI:** 10.3389/fgene.2026.1904219

**Published:** 2026-10-16

**Authors:** Mohammad Ameen Ishqair, Abbas K. H. Masalma, Mohanad Saleh, Fawaz Awad, Aliaa Khalili, Anas Ameen Ishqair, Somaya Salah, Abed-Rahman N. Saleh

**Affiliations:** 1 Medical Research Club, Faculty of Medicine, Al-Quds University, Jerusalem, Palestine; 2 Faculty of Medicine, Lincoln University College, Petaling Jaya, Malaysia; 3 Palestine Polytechnic University, Hebron, Palestine; 4 Dura Governmental Hospital, Department of Internal Medicine., Hebron, Palestine; 5 Department of Molecular Genetics Laboratory, Istishari Arab Hospital, Ramallah, Palestine; 6 Department of Hematology and Oncology, Dura Governmental Hospital, Hebron, Palestine; 7 Genetic Unit, Palestine Medical Complex, Ramallah, Palestine; 8 Genetic Unit, Palestine Red Crescent Society Hospital, Hebron, Palestine; 9 Faculty of Medicine, Hebron University, Hebron, Palestine

**Keywords:** ENT3 deficiency, hypogammaglobulinemia, nephrolithiasis, SLC29A3, SLC29A3-related disorder spectrum

## Abstract

**Background:**

SLC29A3-related disorder spectrum encompasses rare autosomal recessive conditions characterized by multisystem involvement, including immunodeficiency, lymphoproliferation, cutaneous findings, endocrine dysfunction, and growth failure. The clinical presentation is highly heterogeneous, often leading to delayed diagnosis, particularly in adults.

**Case presentation:**

A 33-year-old Palestinian woman born to consanguineous parents presented with a lifelong history of recurrent severe respiratory infections, growth failure (short stature and microcephaly), recurrent nephrolithiasis, chronic vomiting, generalized hirsutism, and bilateral hallux valgus. Laboratory evaluation revealed marked hypogammaglobulinemia (IgG <320 mg/dL, IgA <25 mg/dL, IgM <25 mg/dL), microcytic anemia, and severe vitamin D deficiency. Imaging confirmed chronic pulmonary small-airway changes and a chronically small right kidney with recurrent stones. Whole-exome sequencing identified a homozygous pathogenic SLC29A3 variant (c.1309G>A; p.Gly437Arg), establishing a diagnosis within the SLC29A3-related disorder spectrum. This case is unique due to its adult presentation, the exceptional severity of the humoral immunodeficiency, and the associated renal manifestations, which are infrequently reported in this spectrum.

**Conclusion:**

This report underscores the importance of considering SLC29A3-related disorder spectrum in adults with multisystem disease. Recognition of this spectrum enables targeted multidisciplinary care, including immunoglobulin replacement therapy and anticipatory surveillance for endocrine and renal complications.

## Introduction

SLC29A3-related disorder spectrum (OMIM #602782) comprises a group of rare autosomal recessive conditions caused by biallelic mutations in the *SLC29A3* gene. This gene encodes the equilibrative nucleoside transporter 3 (ENT3) (Kang et al., 2015), which is essential for lysosomal and mitochondrial nucleoside trafficking. Historically, these disorders were described as distinct entities, including H syndrome, Faisalabad histiocytosis, pigmented hypertrichosis with insulin-dependent diabetes (PHID), and familial Rosai-Dorfman disease (Morgan et al., 2010). They are now recognized as a single clinical spectrum characterized by overlapping features such as histiocytic infiltration, lymphadenopathy, hyperpigmentation, hypertrichosis, and endocrinopathies (Molho-Pessach et al., 2008; Molho-Pessach et al., 2014; Molho-Pessach and Zlotogorski 2016).

The clinical phenotype of SLC29A3-related disorder spectrum is remarkably diverse, with significant inter- and intra-familial variability. While pediatric presentations are most common, adult cases provide critical insights into the long-term progression of the disease. This report describes an adult patient with a recurrent homozygous pathogenic variant (p.Gly437Arg) who presented with a unique combination of severe hypogammaglobulinemia and recurrent nephrolithiasis, further expanding the known clinical spectrum. (Al-Haggar et al., 2015; Bakhchane et al., 2016; Rezaie et al., 2024). These support clinical/genetic heterogeneity. Farooq et al. (2012) in adjacent mutation-spectrum background, not for G437R founder effect.

## Methods

### Patient recruitment and consent

The patient was evaluated at Dura Governmental Hospital in Hebron, Palestine. Informed consent was obtained for the clinical evaluation, genetic testing, and publication of this report, including anonymized clinical images.

### Clinical and laboratory evaluation

A comprehensive clinical assessment was performed, including physical examination and a review of medical history. Laboratory investigations included a complete blood count, serum immunoglobulin levels, metabolic panel, and liver function tests. Imaging studies consisted of chest computed tomography (CT) and renal ultrasonography.

### Genetic analysis

Whole-exome sequencing (WES) was performed using the TruSeq Capture Exome Kit on an Illumina platform. Bioinformatics analysis included alignment to the hg38 reference genome, variant calling via GATK, and annotation using ANNOVAR. Variant interpretation was conducted according to the American College of Medical Genetics and Genomics (ACMG) guidelines. The identified homozygous variant was confirmed by Sanger sequencing. Copy-number variant (CNV) analysis was performed using an internally validated pipeline, which yielded no additional significant findings.

### Case presentation

A 33-year-old Palestinian woman, the offspring of first-degree cousin parents, presented with a lifelong history of multisystemic manifestations. Her medical history was notable for recurrent severe lower respiratory tract infections requiring multiple hospitalizations since childhood. She also reported chronic vomiting and recurrent nephrolithiasis, having previously undergone ureteroscopic lithotripsy and double-J stent placement.

On physical examination, she exhibited significant growth failure with a height and weight below the 3rd percentile for her age. She had microcephaly, dysmorphic facial features, long and dense eyelashes, and generalized hirsutism. Skeletal findings included bilateral hallux valgus and mild flexion contractures of the fingers. Her cognitive development was preserved (Figure 1).

**FIGURE 1:**
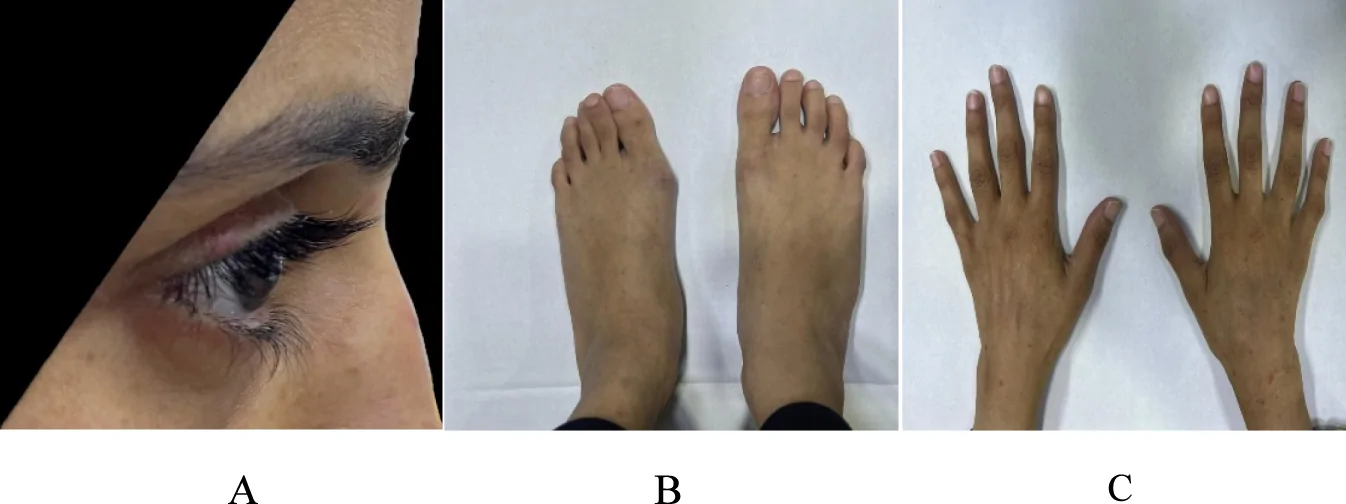
Clinical features of the patient showing **(A)** long and dense eyelashes, **(B)** bilateral hallux valgus deformity of the toes, and **(C)** flexion contractures of the fingers.

Laboratory investigations revealed severe hypogammaglobulinemia, with IgG <320 mg/dL (reference: 700–1600 mg/dL), IgA <25 mg/dL (reference: 70–400 mg/dL), and IgM <25 mg/dL (reference: 40–230 mg/dL). Hematologic evaluation showed microcytic anemia (hemoglobin 10.2 g/dL, MCV 71.9 fL) associated with iron deficiency (iron 29 μg/dL, ferritin 23.5 ng/mL). Liver function tests showed mildly elevated transaminases (ALT 52 U/L, AST 48 U/L; reference <40 U/L). Endocrine evaluation confirmed severe vitamin D deficiency (25-OH vitamin D 7.5 ng/mL) with secondary hyperparathyroidism. Diabetes screening, including fasting blood glucose and HbA1c, was normal at the time of presentation. Key laboratory findings are summarized in Table 1.

**TABLE 1:** Summary of key laboratory findings (abnormal values are bolded and marked with*).

Parameter	Value	Reference range
Immunology		
IgA	<25 mg/dL*	70-400 mg/dL
IgG	<320 mg/dL*	700-1600 mg/dL
IgM	<25 mg/dL*	40-230 mg/dL
Hematology		
Hemoglobin (HGB)	10.2 g/dL*	12.0-16.0 g/dL
Mean Corpuscular Vol. (MCV)	71.9 fL*	80-100 fL
Iron Studies		
Iron	29 µg/dL*	50-170 µg/dL
Ferritin	23.5 ng/mL	15-150 ng/mL
TIBC		
Endocrinology		
Vitamin D (25-OH)	7.5 ng/mL*	30-100 ng/mL
Liver Function		
ALT/AST	Mildly elevated*	
Parameter	Value	Reference Range

(Abnormal values are bolded and marked with *).

Chest CT demonstrated chronic post-infectious small-airway changes, minimal bronchiectasis, and segmental consolidation in the right middle lobe. Abdominal imaging revealed right ureteric stones (8–9 mm) with associated hydronephrosis and a chronically small right kidney (Figures 2–5).

**FIGURE 2:**
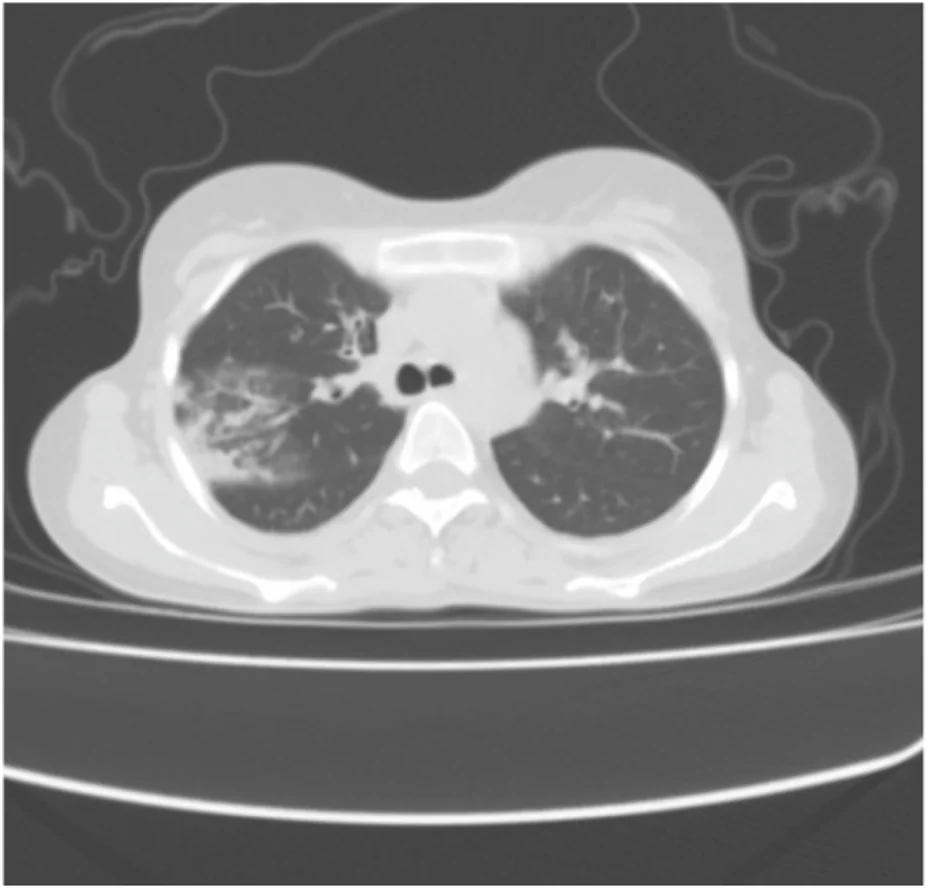
Recurrent or residual bronchopneumonia and segmental consolidations in the right middle lobe.

**FIGURE 3:**
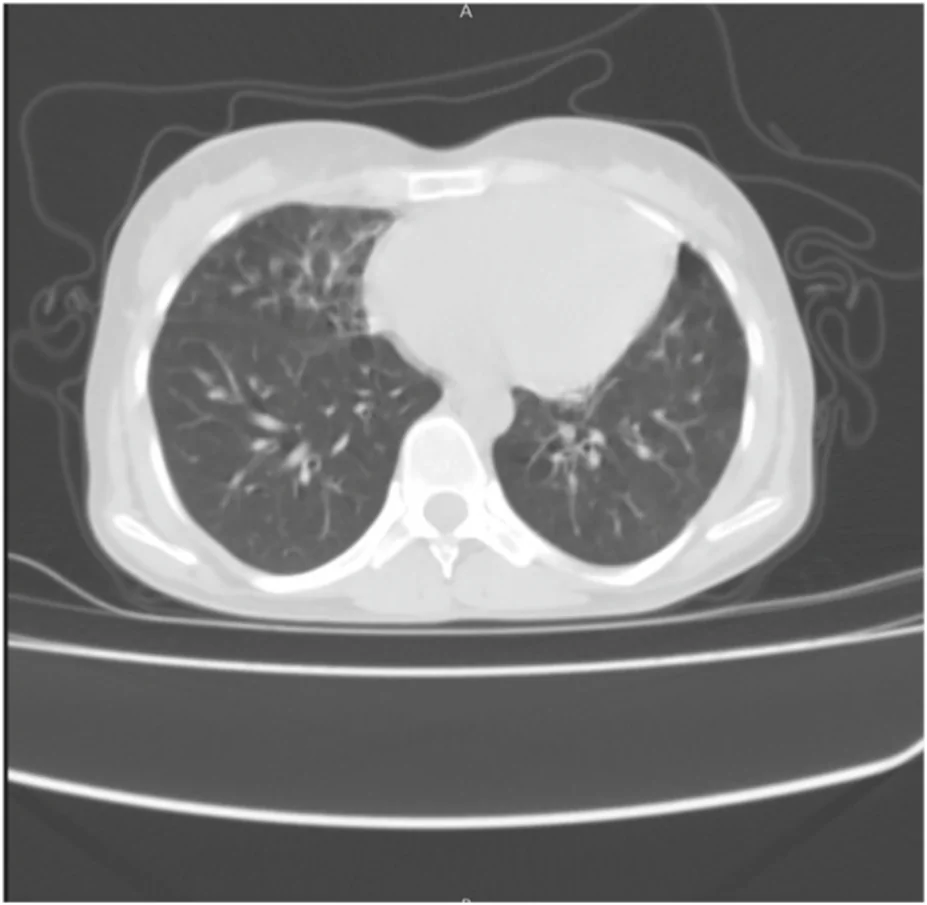
Minimal bronchiectasis and atelectasis.

**FIGURE 4:**
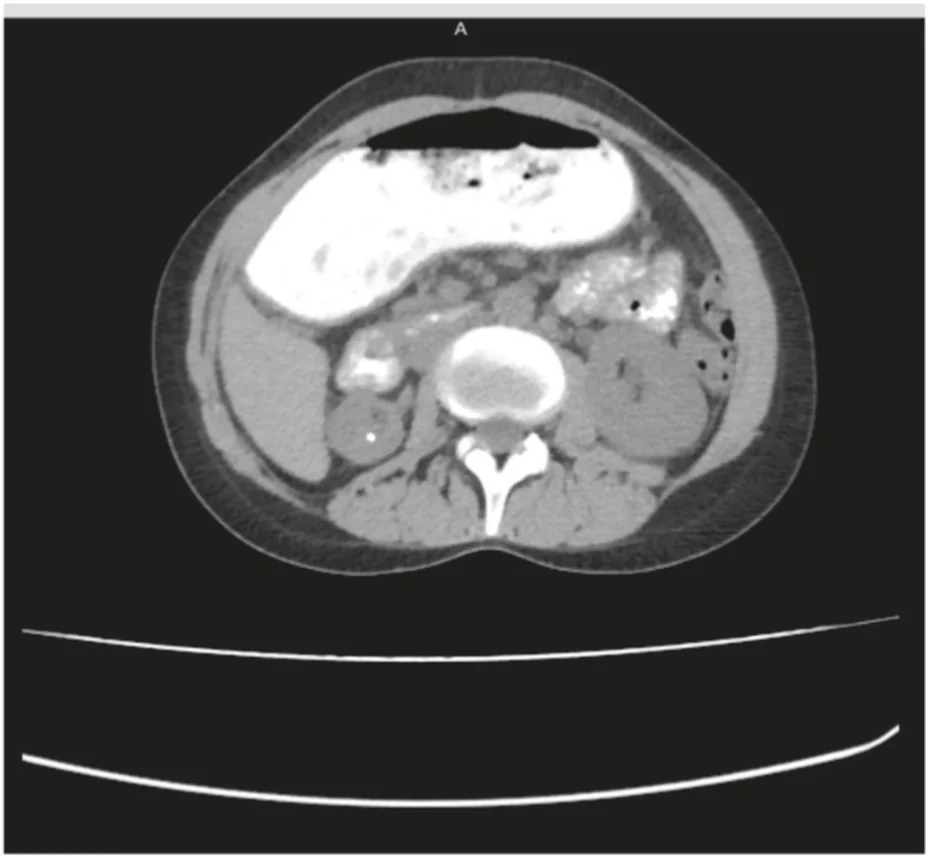
Significant right ureteric obstruction (8–9 mm stones) with mild hydronephrosis.

**FIGURE 5:**
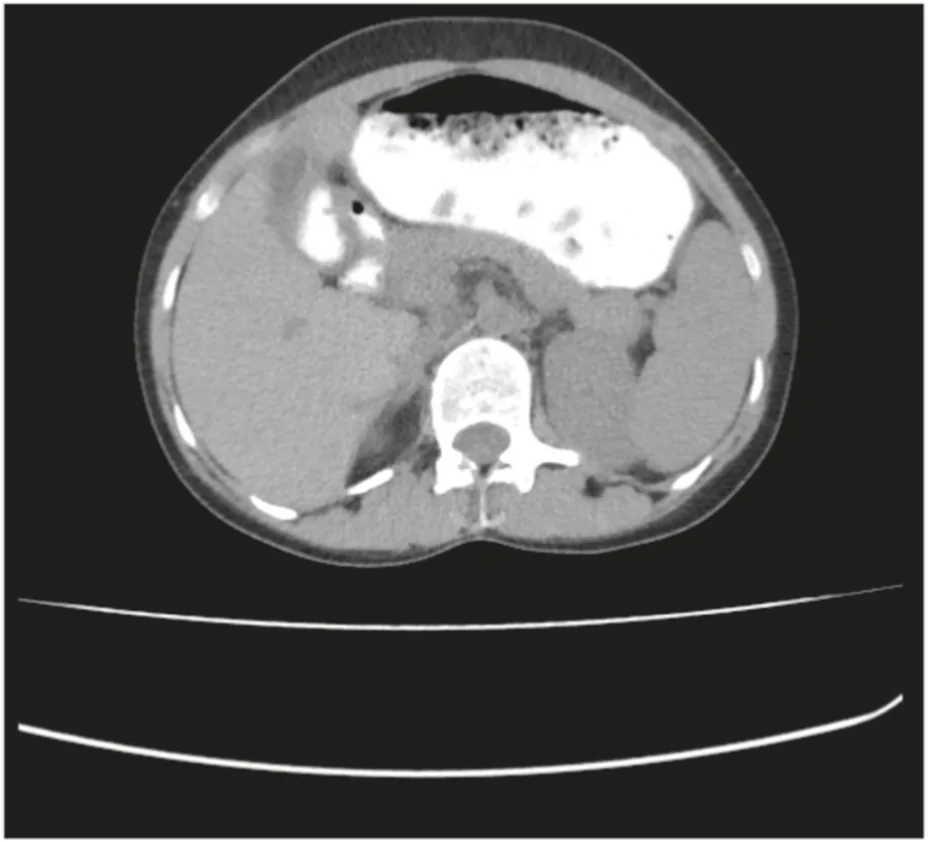
early signs of cholecystitis.

Genetic testing via WES identified a homozygous missense variant in *SLC29A3*: c.1309G>A (p.Gly437Arg). This variant was classified as Pathogenic according to ACMG criteria (PS1, PM2, PP3). The variant was confirmed in the proband by Sanger sequencing. Parental segregation analysis was not performed as the parents were unavailable for testing.

Following the diagnosis, a multidisciplinary management plan was initiated. Immunoglobulin replacement therapy (IVIG) was recommended and initiated, leading to a reduction in the frequency of respiratory infections during the initial 6 months of follow-up. The patient also received vitamin D supplementation and was referred to nephrology for the management of recurrent stones and to endocrinology for ongoing diabetes surveillance.

## Discussion

This case highlights the clinical complexity of SLC29A3-related disorder spectrum and its potential for adult presentation with severe multisystem involvement. The p.Gly437Arg (G437R) variant identified in our patient is a known pathogenic allele frequently reported in Palestinian and Arab populations, In the original gene-discovery study, G437R was identified in several geographically clustered Arab families; haplotype analysis showed a shared disease-associated haplotype among the analyzed G437R families C–F, consistent with a shared origin of the variant in those families (Molho-Pessach et al., 2008). Despite carrying a recurrent variant, our patient’s phenotype is notable for several reasons.

First, the severity of the immunologic phenotype is exceptional. While immunodeficiency and recurrent infections are recognized features of the SLC29A3-related disorder spectrum, the degree of hypogammaglobulinemia observed here—with nearly undetectable IgA and IgM and severely low IgG—is unusual. This raises the possibility that ENT3 deficiency may influence B-cell maturation or function, although the underlying mechanism remains unclear. The clinical improvement following IVIG initiation underscores the importance of early immunologic screening in these patients.

Second, the associated renal manifestations, specifically recurrent nephrolithiasis and a small kidney, are noteworthy. Renal involvement has been reported in approximately 6% of cases within the SLC29A3-related disorder spectrum, including hydronephrosis and renal asymmetry (Bolze, 2012). Our case adds to this evidence, suggesting that renal surveillance should be part of the standard multidisciplinary follow-up. It remains unclear whether these findings are a direct consequence of ENT3-related metabolic dysfunction or secondary to chronic inflammation and infection.

Third, the adult presentation of this case emphasizes the need for clinicians to maintain a high index of suspicion for rare genetic disorders in adults with longstanding, unexplained multisystem symptoms. The combination of growth failure, skeletal anomalies, and immunodeficiency should prompt genetic evaluation even in the absence of the “classic” cutaneous hyperpigmentation often seen in H syndrome.

Comparison with previously reported cases of the G437R variant reveals significant phenotypic heterogeneity (Table 2). While some patients present with predominant cutaneous and endocrine features, others, like our patient, exhibit a more severe immunologic and skeletal phenotype. This variability suggests the influence of genetic modifiers or environmental factors.

**TABLE 2:** Short comparison with reported cases.

Feature	Typical SLC29A3 spectrum	This patient	Comment	Feature
Cutaneous hyperpigmentation/ hypertrichosis	Common	Present	Typical	Cutaneous hyperpigmentation/ hypertrichosis
Endocrinopathy (IDDM, GH deficiency)	Frequent	Vitamin D deficiency; no diabetes reported to date	Diabetes risk — ongoing surveillance needed	Endocrinopathy (IDDM, GH deficiency)
Growth failure / short stature	Frequent	Present (height <3rd centile)	Typical	Growth failure / short stature
Immunodeficiency	Variable	Marked hypogammaglobulinemia; recurrent severe chest infections	More pronounced humoral deficiency	Immunodeficiency
Lymphadenopathy/ histiocytosis	Common	No prominent lymphadenopathy reported	Phenotypic variability	Lymphadenopathy/ histiocytosis
Skeletal anomalies	Reported	Bilateral hallux valgus; long toes	Typical	Skeletal anomalies
Renal involvement	Occasional	Recurrent nephrolithiasis; small right kidney	Requires nephrology follow‑up	Renal involvement

## Limitations

This study is limited by being a single case report. Parental segregation analysis was not performed. Functional studies of ENT3 activity were also unavailable. Importantly, the available immunologic evaluation was limited to quantitative immunoglobulin measurements; extended immune phenotyping, including lymphocyte subsets and vaccine antibody responses, was not available at the time of diagnosis. Similarly, a detailed renal metabolic evaluation, including 24-h urine collection for stone risk factors or stone composition analysis, was not available. Future studies are needed to determine if nephrolithiasis is a primary metabolic manifestation of ENT3 deficiency or an incidental finding.

## Conclusion

This case expands the clinical and demographic spectrum of SLC29A3-related disorder spectrum, highlighting a rare adult presentation with severe humoral immunodeficiency and renal manifestations. Early genetic diagnosis using WES is crucial for establishing a unifying diagnosis and guiding multidisciplinary management, including immunoglobulin replacement therapy, which may substantially reduce infection-related morbidity, and vigilant surveillance for multisystem complications.

## Data Availability

The datasets presented in this article are not readily available because of ethical and privacy restrictions. Requests to access the datasets should be directed to the corresponding author.
